# Dynamic ultrasound for evaluating the adequacy of median nerve decompression following minimally invasive carpal tunnel release: technical innovation and case study

**DOI:** 10.1016/j.heliyon.2023.e13107

**Published:** 2023-01-19

**Authors:** Chien-Hua Chen, Fu-Shan Jaw, Jia-Zhen Hu, Wei-Ting Wu, Ke-Vin Chang

**Affiliations:** a Department of Biomedical Engineering, National Taiwan University, Taipei, Taiwan; b Clive Chen Clinic, Taichung, Taiwan; c Department of Physical Medicine and Rehabilitation, National Taiwan University Hospital, College of Medicine, National Taiwan University, Taipei, Taiwan; d Department of Physical Medicine and Rehabilitation, National Taiwan University Hospital, Bei-Hu Branch, Taipei, Taiwan; e Center for Regional Anesthesia and Pain Medicine, Wang-Fang Hospital, Taipei Medical University, Taipei, Taiwan

**Keywords:** Carpal tunnel syndrome, Median nerve, Sonography, Surgery, Ultrasound, CTS, carpal tunnel syndrome

## Abstract

**Background:**

Minimally invasive carpal tunnel release has recently emerged as the primary surgical approach for recalcitrant carpal tunnel syndrome. A major concern related to surgical failure with this technique is the incomplete release of the flexor retinaculum.

**Case presentation:**

We developed a technique using dynamic ultrasound for evaluating the adequacy of median nerve decompression following minimally invasive carpal tunnel release. This novel imaging method was applied to two patients who showed significant symptom relief after the intervention. This case study also provides details of the dynamic ultrasound protocol and highlights the advantages of this technique.

**Conclusion:**

Dynamic ultrasound imaging can be used to confirm the completeness of carpal tunnel decompression**.** A large-scale prospective trial should be conducted to validate whether additional dynamic ultrasound examination can improve the outcome of minimally invasive carpal tunnel release.

## 1 Introduction

Carpal tunnel syndrome (CTS) is the most common entrapment neuropathy. The prevalence of CTS has been reported between 1.55 and 14.4% [1,2]. The risk of CTS is higher in patients with diabetes, obesity, rheumatoid arthritis, and carpal osteoarthritis [2]. CTS is conventionally diagnosed by the electrophysiological study, and recently ultrasound imaging has been considered a valid diagnostic tool [1]. Non-surgical treatments for CTS include oral medication, bracing, therapeutic exercise, physical modalities, and injections of corticosteroid, 5% dextrose, and platelet-rich plasma [[3], [4], [5], [6]]. Surgical interventions are reserved for cases that do not respond to conservative managements or those with moderate-to-severe CTS. Minimally invasive carpal tunnel release has recently emerged as a mainstream treatment approach because of advantages such as earlier recovery and lower recurrence rates than the standard technique using a limited longitudinal incision [7]. Furthermore, ultrasound imaging can be used to guide the knife during surgery [8]. However, a primary concern associated with minimally invasive carpal tunnel release is incomplete decompression, which leads to unsatisfactory post-surgical symptomatic relief [9]. The aim of the case study was to present a novel application of ultrasound imaging to confirm the adequacy of median nerve decompression after minimally invasive carpal tunnel release. Informed consent was obtained from the patients included in this report.

## 2 Case description

### 2.1 Case 1

A 39-year-old female engineer had been experiencing left-hand numbness for four years. She denied a history of systemic diseases such as diabetes mellitus. Conservative treatments, including physical therapy and medication, were attempted, but resulted in no significant improvement. She also had received corticosteroid perineural injection but the effect was limited. During her first clinic visit, her body mass index was 25.7 kg/m^2^. The diet habit, life style and genetic factors were not contributory to her CTS. Physical examination revealed diminished pinprick sensation over the left first to third fingers and visible thenar muscle atrophy. The Tinel's sign was positive. The visual analogue scale (VAS) score for pain at night was 7 based on a scale of 10 cm. The Boston questionnaire symptom severity scale score was 32, and the functional status scale score was 9 (Supplementary file). Median nerve neuropathy at the wrist level (moderate degree) was confirmed by a subsequent electrophysiological study (Table 1) [10]. The patient underwent minimally invasive carpal tunnel release, and dynamic ultrasound was used to confirm whether the median nerve was adequately decompressed during the procedure. One month later, the Boston symptom severity scale and functional status scale scores decreased to 11 and 8, respectively. She experienced complete relief from the numbness, and the VAS score remained less than 3 thereafter.

**Table 1:** Clinical courses of the two patients.

	Case 1	Case 2
Age (years)	39	56
Gender	Female	Female
Body mass index (kg/m^2^)	25.7	20.8
Diabetes mellitus	(−)	(−)
Symptoms	Diminished pinprick sensation over the left first to third fingers; visible thenar muscle atrophy.	Blurred sensation of light touch over the tips of the left first to third fingers
Preoperative electrophysiological findings	Moderate prolonged distal sensory and motor latency	Moderate prolonged distal sensory and motor latency
Preoperative visual analogue scale of pain at night	7	6
Preoperative Boston questionnaire symptom severity scale score	32	28
Preoperative Boston questionnaire functional status scale score	9	16
Postoperative electrophysiological findings	Normal distal sensory and motor latency	Mild prolonged distal sensory and motor latency
Postoperative visual analogue scale of pain at night	3	2
Postoperative Boston questionnaire symptom severity scale score	11	21
Postoperative Boston questionnaire functional status scale score	8	12

### 2.2 Case 2

A 56-year-old female office worker presented with a three-year history of left-hand numbness. She had a normal body mass index (20.8 kg/m^2^) and denied any chronic diseases. Physical modalities such as transcutaneous electric stimulation and low-power laser only partially relieved her discomfort. Her diet habit, life style and genetic factors did not contribute to her CTS. During the physical examination, the sensation of light touch became blurred over the tips of the left first to third fingers with a positive Tinel's sign. The Boston questionnaire symptom severity scale score was 28, and the functional status scale score was 16.

An electrophysiological study confirmed moderate median nerve compression at the carpal tunnel level (Table 1) [10]. A minimally invasive carpal tunnel release was performed on the affected wrist under intraoperative dynamic ultrasound monitoring to ensure complete decompression. One month later, the Boston symptom severity scale and functional status scale scores reduced to 21 and 12, respectively. Her VAS score at night also decreased from 6 preoperatively and 2 postoperatively.

### 2.3 Technique reports

**Fig. 1:**
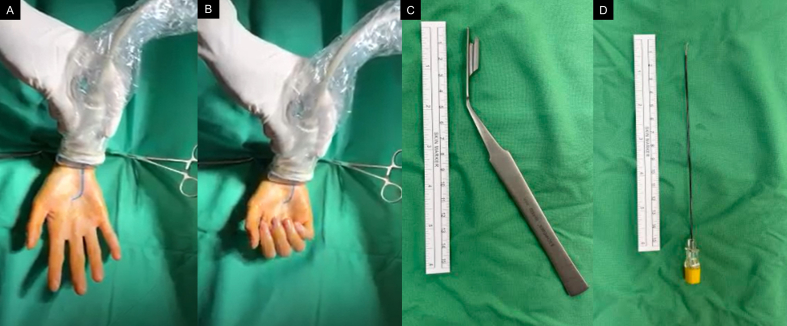
Ultrasound evaluation of the carpal tunnel during finger extension (A) and flexion (B), with the blue line on the palm indicating the superficial palmar arch of the ulnar artery. The carpal tunnel knife for minimally invasive carpal tunnel release (C). The blunt-tipped needle for confirmation of the completeness of decompression (D). (For interpretation of the references to colour in this figure legend, the reader is referred to the Web version of this article.)

**Fig. 2:**
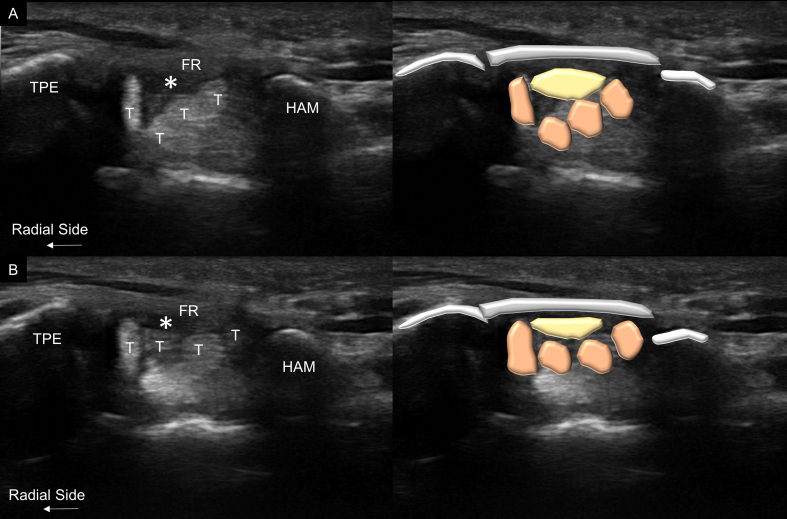
Ultrasound imaging and schematic drawing of the carpal tunnel outlet before the surgery in the neutral resting position (A) and finger extension (B). The median nerve (asterisks) appeared flattened and compressed between the flexor retinaculum (FR) and flexor tendons (T) when the fingers were extended. TPE, trapezium; HAM, hamate.

The intervention was performed by a neurosurgeon who also had more than 10 years in practicing neuromuscular ultrasound. Both patients were given acetaminophen for post-operative pain control. The post-intervention follow-up was set at one month later. During minimally invasive carpal tunnel release, the patients were positioned in the supine position. The operative field was sterilized using a Betadine Surgical Scrub Solution. The ultrasound probe was draped with an aseptic cover. Subsequently, the carpal tunnel was scanned with a high-frequency linear transducer (10–18 MHz, MyLab 25 gold; Esaote S.p.A, Genova, Italy). The median nerve and adjacent finger flexor tendons within the carpal tunnel were evaluated using ultrasound axial imaging to determine the presence of anatomical variants (such as persistent median arteries or bifid median nerves). Dynamic motion of the median nerve was examined by asking the patient to flex (Fig. 1A) and extend (Fig. 1B) the fingers. The upward migration of the median nerve was limited by the flexor retinaculum (Fig. 2A and B and Video 1). The length of the flexor retinaculum was evaluated longitudinally to determine the distance to which the carpal tunnel knife should be advanced during surgery.

Supplementary video related to this article can be found at https://doi.org/10.1016/j.heliyon.2023.e13107

The following is the supplementary data related to this article:

Video 1 1 Video 1

A transverse incision of approximately 10 mm was then performed at the ulnar side of the palmaris longus over the distal wrist crease after local anesthesia with 5 mL of 1% lidocaine. The wound was deepened with blunt dissection until the flexor retinaculum was observed. A small scissor was used to make an opening of the flexor retinaculum, and the median nerve was identified under direct visualization. A blunt-tipped needle was inserted through the opening to investigate the route for carpal tunnel release.

The carpal tunnel knife (Fig. 1C) was then inserted with one tip above and the other tip below the flexor retinaculum. The knife was advanced toward the ring finger and kept away from the marked courses of the ulnar artery and superficial palmar arch. The carpal tunnel knife was advanced forward by approximately 3–4 cm to separate the flexor retinaculum. After cutting, the knife was removed.

**Fig. 3:**
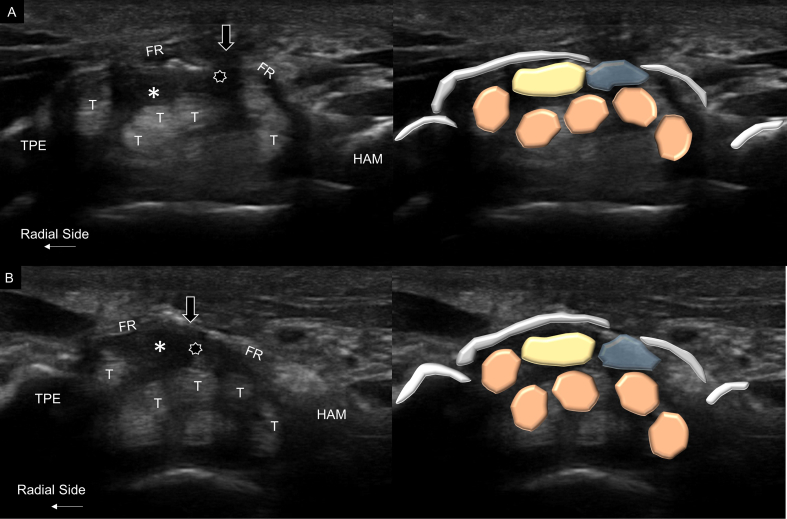
Ultrasound imaging and schematic drawing of the carpal tunnel outlet after surgery in the neutral resting position (A) and finger flexion (B). The median nerve (asterisks) appeared to be pushed upward by the finger flexor tendons (T) and bulged through the gap (black arrows) on the flexor retinaculum (FR) when the fingers were flexed. TPE, trapezium; HAM, hamate. Stars, synovial tissue.

An ultrasound probe was used to confirm the adequacy of median nerve decompression. In this evaluation, the transducer was first placed in the axial plane at the carpal tunnel outlet. The patient was asked to flex and extend the five fingers with the wrist slightly extended. During finger movements, the median nerve was pushed upward by the adjacent/underlying flexor digitorum superficialis and profundus tendons. Complete decompression was defined by the visualization of a full-thickness gap over the flexor retinaculum with the median nerve migrating more superficially than the imaginary line linking the trapezium and hamate (Fig. 3A and B and Video 2).

**Fig. 4:**
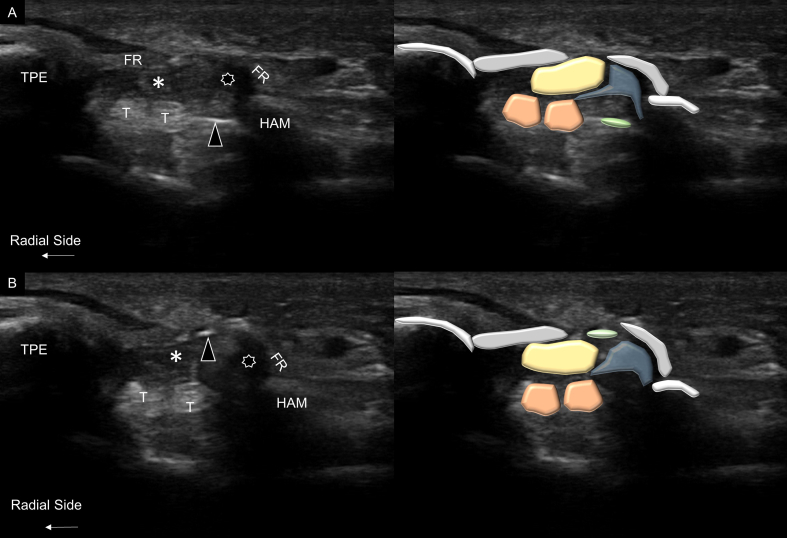
The blunt-tipped needle (black arrowheads) was inserted into the opened carpal tunnel outlet (A) and gradually moved upward (B) to confirm the completeness of surgical release. TPE, trapezium; HAM, hamate; FR, flexor retinaculum; T, flexor tendons; stars, synovial tissue; asterisks, median nerve.

Subsequently, the blunt-tipped needle (Fig. 1D) was introduced again into the carpal tunnel. Under ultrasound guidance, the needle was moved upward to check the continuity of the flexor retinaculum. Decompression was considered complete if the needle could be seen moving from the carpal tunnel to the subcutaneous layer through the gap on the flexor retinaculum (Fig. 4A and B and Video 3).

Supplementary video related to this article can be found at https://doi.org/10.1016/j.heliyon.2023.e13107

The following are the supplementary data related to this article:

Video 22

Video 3 3 Video 3

## 3 Discussion

Ultrasound imaging has been widely used in the diagnosis and management of peripheral nerve entrapment syndrome. According to a recent meta-analysis [11], the performance of the ultrasound-measured nerve cross-sectional area was comparable to electrophysiological evaluations for the diagnosis of CTS. Furthermore, another systematic review [12] pointed out that the effectiveness of perineural injection for median nerves was greater when ultrasound guidance was used instead of the landmark-based approach. The combination of ultrasound imaging with percutaneous minimally invasive surgeries enables the localization of important neurovascular structures before intervention, leading to a reduced risk of nerve injury [13]. Although safety has been substantially improved with the use of ultrasound guidance, studies investigating the ability of ultrasound imaging to evaluate complete decompression of the carpal tunnel are limited.

In a previous study, incomplete release of the carpal tunnel accounted for 28.6% of the failed cases after carpal tunnel release [14]. Static ultrasound shows limitations in recognizing the completeness of carpal tunnel release, because the dissected tissues of the flexor retinaculum may overlap when the transducer is applied. In this regard, the potential for facilitating dynamic examinations is a major benefit of ultrasound imaging [[15], [16], [17], [18]]. When patients are asked to flex and extend the fingers, the median nerve glides over the finger flexors with changes in the nerve shape. Excursion of the median nerve has been found to be reduced in patients with CTS [19,20]. Furthermore, a recent cadaveric study highlighted that the median nerve paraneural sheath had connections with adjacent myofascial structures [21]. The paraneural adipose tissues also reduced from the proximal to distal levels, which rendered the median nerve to be easily tethered by the connective tissues at the carpal tunnel. In participants with CTS, ultrasound was shown helpful in the detection of decreased nerve displacement. The treatment failure following surgical release could be derived from the continuity between the paraneural sheath and epimysial fasciae of the neighboring tendons, which might be recognized by dynamic ultrasonographic imaging.

The benefits of our proposed techniques can be highlighted in the following points. First, we defined the completeness of carpal tunnel release by using dynamic ultrasound, which allows detailed evaluation of fiber continuity of the flexor retinaculum. Incomplete decompression during minimally invasive carpal tunnel release is not a rare finding [22]. When advancing the carpal tunnel knife, the superficial portion of the flexor retinaculum may only be partially dissected, with the remaining fibers remaining attached to the overlying palmar aponeurosis. Second, dynamic ultrasound prevented the accidental injury of the nearby vasculature during intervention. Since only a small incision was created near the carpal tunnel inlet during the minimally invasive carpal tunnel release, direct visualization of the retinaculum over the carpal tunnel outlet was not possible. However, when performing the procedure, the primary concern is the superficial palmar arch [23], which is located slightly distal to the flexor retinaculum. Therefore, the surgeon may choose to avoid advancing the knife too distally, leaving the distal flexor retinaculum partially dissected and causing incomplete decompression.

Our ultrasound technique showed several advantages and clinical implications. First, the definition of completeness of carpal tunnel release was objective. Second, dynamic ultrasound imaging was performed to compare the changes in median nerve mobility before and after the surgery. Third, once the carpal tunnel is found to be incompletely released, the surgeon can further dissect the flexor retinaculum to achieve complete decompression.

On the other hand, static ultrasound imaging is useful for measuring the cross-sectional area of the median nerve as well as identifying lesions that cause the compression of the median nerve [1]. However, the mobility of the median nerve requires dynamic ultrasound to validate. Asking the patients to flex and extend the fingers, the reciprocal movement between the median nerve and finger flexors can be easily recognized by B-mode imaging. Using the speckle tracking algorithm, the investigators can further obtain the quantitative data of nerve excursion [24].

The main limitation of this technical note is the number of cases. First, only two patients were included in the study. Thus, the benefits of intraoperative ultrasound scanning in patients undergoing minimally invasive surgery require further validation. Furthermore, due to the small sample, it was hard to know the exact percentage of incomplete release following the use of our novel approach, waiting more large-scaled trials to explore. In the present study, some details of outcome assessment (such as nerve displacement on ultrasound imaging after carpal tunnel release) were not prospectively collected. As a pilot study, its value lay on the novel ultrasound guided evaluation technique, whereas its true benefit requires more future quantitative data to investigate. Second, we did not perform 2-point discrimination for both cases, which should be incorporated in the pre/post-operative assessment in subsequent research. Third, the speckle tracking algorithm, a technique commonly applied on echocardiography, might be useful for evaluating vectors ad velocity of the median nerve during dynamic examination and can be incorporated in future studies to provide more quantitative data [24].

## 4 Conclusion

Dynamic ultrasound imaging can be used to confirm the completeness of carpal tunnel decompression. A prospective study should be conducted to examine whether additional dynamic ultrasound dynamic examination can improve the outcome of minimally invasive carpal tunnel release.

## Author contribution statement

All authors listed have significantly contributed to the investigation, development and writing of this article.

## Funding statement

This work was funded by National Taiwan University Hospital, Bei-Hu Branch (funding number: 11202); Ministry of Science and Technology (MOST 106-2314-B-002-180-MY3 and 109-2314-B-002-114-MY3); and the Taiwan Society of Ultrasound in Medicine.

## Data availability statement

Data will be made available on request.

## Declaration of interest’s statement

The authors declare no competing interests.
