# Supplementary material for: Dynamic ultrasound for evaluating the adequacy of median nerve decompression following minimally invasive carpal tunnel release: technical innovation and case study
Source: Heliyon. 2023 Jan 19;9(1):e13107. doi: 10.1016/j.heliyon.2023.e13107 (PMC9880394; doi:10.1016/j.heliyon.2023.e13107)
Supplement: Multimedia component 4 [file mmc4.pdf]

Queensland Government

Metro North Hospital and Health Service

BOSTON CARPAL TUNNEL QUESTIONNAIRE SEVERITY SCALE

(Affix patient identification label here)

URN:

Family Name:

Given Names:

Address:

Date of Birth: Sex: ☐ M ☐ F ☐ I

Carpal Tunnel Syndrome Severity Scale:

Adapted from 'The Boston Carpal Tunnel Questionnaire', Levine et al, 1993

This is a short questionnaire used to help monitor the progress of your carpal tunnel syndrome symptoms in your **hand**. The following questions refer to your **hand** on a typical day in the past 2 weeks. Please TICK only one answer per question.

How severe is the hand or wrist pain that you have at night?

- ☐ I do not have hand or wrist pain at night 1
- ☐ Mild pain 2
- ☐ Moderate pain 3
- ☐ Severe pain 4
- ☐ Very severe pain 5

How often did hand or wrist pain wake you up during a typical night in the past two weeks?

- ☐ Never 1
- ☐ Once 2
- ☐ Two or three times 3
- ☐ Four or five times 4
- ☐ More than five times 5

Do you typically have pain in your hand or wrist during the daytime?

- ☐ I never have pain during the day 1
- ☐ I have mild pain during the day 2
- ☐ I have moderate pain during the day 3
- ☐ I have severe pain during the day 4
- ☐ I have very severe pain during the day 5

How often do you have hand or wrist pain during the daytime?

- ☐ Never 1
- ☐ Once or twice a day 2
- ☐ Three to five times a day 3
- ☐ More than five times a day 4
- ☐ The pain is constant 5

How long, on average, does an episode of pain last during the daytime?

- ☐ I never get pain during the day 1
- ☐ Less than 10 minutes 2
- ☐ 10 to 60 minutes 3
- ☐ Greater than 60 minutes 4
- ☐ The pain is constant throughout the day 5

Do you have numbness (loss of sensation) in your hand?

- ☐ No 1
- ☐ I have mild numbness 2
- ☐ I have moderate numbness 3
- ☐ I have severe numbness 4
- ☐ I have very severe numbness 5

DO NOT WRITE IN THIS BINDING MARGIN
Do not reproduce by photocopying
All clinical form creation and amendments must be conducted through Health Information Services

MR A 8505
V2.00 - 09/2016
Locally Printed
00201:08505

Queensland Government

Metro North Hospital and Health Service

BOSTON CARPAL TUNNEL QUESTIONNAIRE SEVERITY SCALE

(Affix patient identification label here)

URN:

Family Name:

Given Names:

Address:

Date of Birth: Sex: ☐ M ☐ F ☐ I

Do you have weakness in your hand or wrist?

☐ No weakness	1
☐ Mild weakness	2
☐ Moderate weakness	3
☐ Severe weakness	4
☐ Very severe weakness	5

Do you have tingling sensations in your hand?

☐ No tingling	1
☐ Mild tingling	2
☐ Moderate tingling	3
☐ Severe tingling	4
☐ Very severe tingling	5

How severe is numbness (loss of sensation) or tingling at night?

☐ I have no numbness or tingling at night	1
☐ Mild	2
☐ Moderate	3
☐ Severe	4
☐ Very severe	5

How often did hand numbness or tingling wake you up during a typical night during the past two weeks?

☐ Never	1
☐ Once	2
☐ Two or three times	3
☐ Four or five times	4
☐ More than five times	5

Do you have difficulty with the grasping and use of small objects such as keys or pens?

☐ No difficulty	1
☐ Mild difficulty	2
☐ Moderate difficulty	3
☐ Severe difficulty	4
☐ Very severe difficulty	5

Clinician use only:

SCORE: __________ (Total Score) / 11 = ___________ (Average Score)

Date:........../........./.........

Completed by *(print name)*:.. Signature:..

Designation:.. Date:........./........./.........

DO NOT WRITE IN THIS BINDING MARGIN

Queensland Government

Metro North Hospital and Health Service

BOSTON CARPAL TUNNEL QUESTIONNAIRE FUNCTIONAL STATUS SCALE

(Affix patient identification label here)

URN:

Family Name:

Given Names:

Address:

Date of Birth: Sex: ☐ M ☐ F ☐ I

On a typical day in the past two weeks, have hand or wrist symptoms caused you to have any difficultly completing the activities listed below?

Please tick one number that best describes your ability to do the activity.

☐ Left hand ☐ Right hand

Activity	No Difficulty	Mild Difficulty	Moderate Difficulty	Severe Difficulty	Cannot do at all due to symptoms
Writing	☐ 1	☐ 2	☐ 3	☐ 4	☐ 5
Buttoning of clothes	☐ 1	☐ 2	☐ 3	☐ 4	☐ 5
Holding a book while reading	☐ 1	☐ 2	☐ 3	☐ 4	☐ 5
Gripping of a telephone handle	☐ 1	☐ 2	☐ 3	☐ 4	☐ 5
Opening of jars	☐ 1	☐ 2	☐ 3	☐ 4	☐ 5
Household chores	☐ 1	☐ 2	☐ 3	☐ 4	☐ 5
Carrying of grocery bags	☐ 1	☐ 2	☐ 3	☐ 4	☐ 5
Bathing and dressing	☐ 1	☐ 2	☐ 3	☐ 4	☐ 5

Clinician use only:

SCORE: .... **(Total Score) / 8 =** .... **(Average Score)**

Completed by *(print name)*: .... Designation: ....

Signature: .... Date: ...... / ...... / ......

DO NOT WRITE IN THIS BINDING MARGIN
Do not reproduce by photocopying
All clinical form creation and amendments must be conducted through Health Information Services

MR A 8508
V1.00 - 09/2016
Locally Printed
00201:08508

BOSTON CARPAL TUNNEL QUESTIONNAIRE FUNCTIONAL STATUS SCALE

收案編號:＿＿＿＿＿＿ ☐第 0 週 ☐ 第 6 週 ☐第 12 週

腕隧道症候群症狀嚴重度及功能量表

下列表格填表為 ☐右手 ☐左手 的評量結果

本問卷的目的，是要瞭解您的腕隧道症候群嚴重程度及功能狀況，共有兩個部分，請您依實際的狀況依序作答。

第一部分：**症狀嚴重程度**

下列問題請依據您過去兩星期內，每天 24 小時中產生的症狀回答
（每一題請圈選一個答案）

**(一)您在半夜時，手或手腕疼痛的程度？**<br>1. ……我的手或手腕沒有出現半夜疼痛。<br>2. ……輕微的疼痛。<br>3. ……中等的疼痛。<br>4. ……嚴重的疼痛。<br>5. ……非常嚴重的疼痛。	**(五)白天出現疼痛，平均每次會持續多久？**<br>1. ……從來沒有。<br>2. ……少於 10 分鐘。<br>3. ……10 到 60 分鐘。<br>4. ……大於 60 分鐘。<br>5. ……整個白天都持續在痛。
**(二)過去兩星期內，您平時一個晚上多常因半夜手或手腕疼痛而醒來？**<br>1. ……從來沒有。<br>2. ……一次。<br>3. ……二次 或 三次。<br>4. ……四次 或 五次。<br>5. ……大於五次。	**(六)您的手會感覺麻木（失去感覺）嗎？**<br>1. ……不會。<br>2. ……輕微感覺麻木。<br>3. ……中等感覺麻木。<br>4. ……嚴重感覺麻木。<br>5. ……非常嚴重感覺麻木。
**(三)您平時在白天會手或手腕疼痛嗎？**<br>1. ……我在白天不會疼痛。<br>2. ……我在白天會輕微疼痛。<br>3. ……我在白天會中度疼痛。<br>4. ……我在白天會嚴重疼痛。<br>5. ……我在白天會非常嚴重疼痛。	**(七)您的手或手腕會無力嗎？**<br>1. ……不會無力。<br>2. ……輕微無力。<br>3. ……中等無力。<br>4. ……嚴重無力。<br>5. ……非常嚴重無力。
**(四)您多常在白天出現手或手腕疼痛?**<br>1. ……從來沒有。<br>2. ……一天 一次或二次。<br>3. ……一天 三次至五次。<br>4. ……一天 大於五次。<br>5. ……整天持續在痛。	**(八)您的手會有刺痛感嗎？**<br>1. ……沒有刺痛感。<br>2. ……輕微刺痛感。<br>3. ……中等刺痛感。<br>4. ……嚴重刺痛感。<br>5. ……非常嚴重刺痛感。

(九)在半夜出現感覺麻木（失去感覺）或刺痛感有多嚴重？

1. ……我沒有出現感覺麻木或刺痛感。
2. ……輕微。
3. ……中等。
4. ……嚴重。
5. ……非常嚴重。

(十)過去兩星期內，您平時一個晚上多常因半夜出現感覺麻木或刺痛感而醒來？

1. ……從來沒有。
2. ……一次。
3. ……二次 或 三次。
4. ……四次 或 五次。
5. ……大於五次。

(十一)您抓握和使用小東西，如鑰匙或筆或拿碗筷，會有困難嗎？

1. ……沒有困難。
2. ……輕微困難。
3. ……中等困難。
4. ……嚴重困難。
5. ……非常嚴重困難。

第二部分：**功能狀態**

在過去兩星期內，因為您手和手腕的症狀，是否使您在執行下列活動時有困難?
請圈選一個最能描述您活動能力的數字。

活動內容	沒有困難	輕微困難	中等困難	嚴重困難	因手或手腕的症狀完全無法執行
寫字	1	2	3	4	5
扣衣服鈕扣	1	2	3	4	5
拿著書本閱讀	1	2	3	4	5
握著電話話筒	1	2	3	4	5
打開寬口的瓶罐	1	2	3	4	5
家事打理	1	2	3	4	5
提購物袋	1	2	3	4	5
洗澡和穿衣服	1	2	3	4	5
